# Dermal Exposure to Heavy Metals in Urban Green Space Soils: A Review of Bioavailability, Toxic Mechanisms, and Precision Risk Assessment

**DOI:** 10.3390/toxics14030236

**Published:** 2026-03-10

**Authors:** Yiping Cheng, Daolei Cui, Zhaolai Guo, Wei Hong, Yue Li, Chin Wei Lai, Ping Xiang

**Affiliations:** 1 Yunnan Key Laboratory of Plateau Wetland Conservation, Restoration and Ecological Services, Institute of Environmental Remediation and Human Health, School of Ecology and Environment, Southwest Forestry University, Kunming 650224, China; chengyiping@swfu.edu.cn (Y.C.); daolei_cui@126.com (D.C.); 18314522882@163.com (Z.G.); 2 Key Laboratory of Endemic and Ethnic Diseases, Ministry of Education, Guizhou Medical University, Guiyang 550004, China; hongwei@gmc.edu.cn; 3 Nanotechnology & Catalysis Research Centre (NANOCAT), Institute for Advanced Studies (IAS), University of Malaya (UM), Kuala Lumpur 50603, Malaysia; liyue@hhtc.edu.cn (Y.L.); cwlai@um.edu.my (C.W.L.); 4 Yunnan Provincial Key Laboratory of Public Health and Biosafety, Kunming 650500, China

**Keywords:** urban soil pollution, bioavailability, in vitro models, transdermal penetration, cutaneous toxicology, precision governance

## Abstract

Urban green spaces (UGSs) provide essential ecological services but also accumulate heavy metals (HMs) in their soils, posing a paradoxical health risk through dermal exposure. Traditional risk assessments, based solely on total HM concentrations, often overestimate threats by ignoring bioavailability (the fraction actually absorbed by organisms) and dynamic skin microenvironment factors. This review synthesizes recent advances to propose a precision assessment framework that integrates bioavailability. The framework advocates for the incorporation of bioaccessibility (the fraction of pollutants dissolved in body fluids)-driven exposure metrics (e.g., physiologically based extraction tests), mechanistic dermal permeation models (e.g., Franz diffusion cells, 3D skin constructs), and population-specific susceptibility factors (e.g., children, individuals with compromised skin). We elucidate how soil properties (pH, organic matter) and metal speciation (e.g., Cr(III)/Cr(VI)) modulate cutaneous uptake, and detail toxicological mechanisms including oxidative stress, ferroptosis/cuproptosis, immunotoxicity, and pigmentation disorders. Case studies reveal heterogeneous HM hotspots in high-traffic and densely populated areas, while in vitro–in vivo extrapolation highlights the potential for misestimation in traditional models. Consequently, we discuss the limitations and future directions of this framework, aiming to shift UGS risk management from over-conservative assessment to bioavailability-based precision governance, thereby supporting the health security of sustainable urban habitats.

## 1. Introduction

Urban green spaces (UGSs) serve as vital ecological infrastructure for modern cities, offering multifaceted benefits ranging from mitigating urban heat island effects to enhancing public psychological well-being and reducing cardiovascular disease prevalence [1]. These ecosystems serve as natural filters for airborne pollutants and carbon sinks, yet their soil matrices concurrently accumulate persistent toxicants. Paradoxically, these urban health sanctuaries are increasingly compromised by heavy metal (HM) accumulation in soils. While these green spaces provide vital ecological benefits, the accumulated heavy metals may pose potential human health risks [2]. Soils adjacent to high-traffic urban corridors exhibit lead (Pb), zinc (Zn), and Copper (Cu) concentrations approximately threefold higher than background levels [3]. This “roadside effect” propagates directly into urban agriculture [4,5]. For instance, Zn concentration in tomato leaves peaks at 144 mg kg^−1^ dry weight (dw) 10 m from the roadway, then plummets to 38 mg kg^−1^ dw at 60 m—a reduction exceeding 70%. Similarly, zucchini leaves show a concomitant decline from 140 to 13.5 mg kg^−1^ dw over the same distance. These data demonstrate that traffic-derived heavy metals decay exponentially with distance but still accumulate significantly in vegetable tissues [6].

The dermal pathway—long underestimated in traditional risk paradigms—constitutes a critical exposure route for urban populations engaging in recreational activities (e.g., gardening, sports, picnicking). Occupational surveys indicate 63% of gardeners recognize dermal contact as a primary exposure vector [7], while children exhibit elevated susceptibility due to frequent hand-to-soil contact and developmentally immature epidermal barriers [8]. Contrary to the historical perception of skin as an impermeable barrier, contemporary evidence confirms its role as a dynamic interface for HMs uptake, particularly under compromised barrier conditions [9]. Resuspended soil particles adhering to sweat-moistened skin further amplify bioavailability, facilitating transdermal migration of contaminants like cadmium (Cd), chromium (Cr), and Pb. Marin et al. demonstrated that sebum-laden sweat enables virtually the entire bioaccessible chromium pool to traverse a reconstructed skin barrier [10], while Larese showed that synthetic sweat oxidizes heavy-metal ions, thereby augmenting their transdermal flux [11]. Beyond systemic toxicity, direct cutaneous insults manifest as dermatoses, including inflammatory disorders (e.g., contact dermatitis, psoriasis) and accelerated skin aging [12].

Conventional skin risk assessment frameworks exhibit two fundamental limitations: (1) Methodological oversimplification: Overreliance on total HM concentrations, ignoring bioavailability modulated by soil physicochemical properties (e.g., organic matter composition reducing urban HM mobility) and metal speciation (e.g., Cr(III) oxidation to carcinogenic Cr(VI) in acidic sweat) [13]. (2) Physiological realism gap: Neglect of dynamic dermal microenvironmental factors, including sweat pH fluctuations (4.0–7.5) altering metal solubility [14], sebum-mediated permeation [15], and barrier integrity variations (e.g., permeability increase in damaged skin) [16]. To address these gaps, this review advances a precision assessment framework integrating: Bioavailability-driven exposure metrics (e.g., bioaccessibility via Physiologically Based Extraction Tests); mechanistic models of dermal permeation (e.g., transcellular vs. shunt diffusion pathways; pathophysiological susceptibility factors (e.g., child-specific behaviors, occupational exposure). By reconciling environmental science with cutaneous toxicology, this work aims to transform UGS risk management—replacing “over-assessment” with economically viable, targeted interventions.

## 2. Literature Search Strategy

This review adopts a systematic approach to summarize the literature on dermal exposure to heavy metals in urban green space soils and related health risk assessment methods. A comprehensive literature search was conducted across multiple electronic databases, including Web of Science, PubMed, and Google Scholar, to cover interdisciplinary research in environmental science, toxicology, and public health.

The search strategy employed a combination of keywords and Boolean operators to refine the results. The core search terms included: (1) environmental contaminants (“heavy metal” OR “trace metal” OR “metalloid”) AND (“urban green space” OR “urban soil”); (2) contamination risk and human health risk assessment (“pollution assessment model” OR “health risk assessment” OR “bioavailability” OR “bioaccessibility”); (3) skin exposure model (“dermal exposure” OR “skin absorption” OR “percutaneous” OR “transdermal”) AND (“reconstructed human epidermis” OR “3D skin model” OR “skin organoid” OR “skin-on-chip” OR “in vitro dermal model”); (4) mechanistic studies of dermal toxicity (“signaling pathway” OR “transcriptomic” OR “proteomic” OR “metabolomic”) AND (“keratinocyte” OR “fibroblast” OR “melanocyte” OR “Langerhans cell” OR “stratum corneum” OR “dermal papilla”). The search was initially focused on the literature published between January 2000 and December 2024, with particular emphasis on seminal works and recent advances (post-2015). A total of 1686 records were retrieved through keyword combination searches.

The retrieved records were screened based on their titles and abstracts. Studies were included if they (1) investigated HM contamination in UGS or similar urban surface soils; (2) examined dermal exposure pathways, bioavailability/bioaccessibility assays, or in vitro/in vivo skin permeation models; or (3) proposed frameworks for health risk assessment. Literature reviews, original research articles, and relevant book chapters were considered. Non-English publications and studies focusing exclusively on ingestion or inhalation pathways without dermal relevance were excluded. Finally, 116 articles were included.

## 3. Heavy Metal Contamination in Urban Green Space Soils and Health Risk Assessment Methodologies

### 3.1. Sources and Multisystem Hazards of Heavy Metal Pollution in Urban Green Spaces

UGSs play a vital role in improving environmental quality and public well-being, yet they are increasingly compromised by HM contamination. The composition of urban soils is highly heterogeneous, comprising not only natural substrates but also anthropogenic materials such as construction backfill and urban waste [17,18]. This complexity complicates contamination assessment and necessitates a detailed understanding of pollution sources and pathways.

The primary anthropogenic sources of HMs in UGSs include: (1) Traffic Emissions—predominantly through abrasion of brake pads and tires in densely urbanized areas—serve as a dominant source of Cr, nickel (Ni), and Pb released into the environment [19]. (2) Industrial Activities—Manufacturing, mining and energy-generation operations [20]. (3) Waste Disposal and Urban Construction—shaped by construction debris, and freight-related goods-movement operations, results in marked accumulation of Cu, Zn and Pb [21,22]. These emissions are transported atmospherically and deposited onto UGSs via dry and wet deposition [23,24]. Consequently, HM pollution exhibits spatial heterogeneity, with higher concentrations typically observed in densely populated urban cores [25,26,27], thereby exposing larger populations to potential health risks. The human health impacts of HM exposure are equally concerning, affecting multiple physiological systems. Neurotoxic metals such as Pb, Cd, As, and Mercury (Hg) have been linked to a spectrum of neurological disorders [28,29]. The immune system is particularly sensitive to HM exposure, with studies demonstrating dose-dependent suppression of critical immune markers [30,31,32].

These findings underscore the complex, multisystem nature of HM toxicity, which necessitates a correspondingly sophisticated and accurate risk assessment framework to inform effective management. However, the predominant traditional assessment paradigms, which will be critically examined in the next section, are fundamentally limited in their capacity to capture this complexity.

### 3.2. Limitations of Traditional Health Risk Assessment Methods for Heavy Metals

Conventional health risk assessments for heavy metals (HMs) in urban soils predominantly rely on total concentration quantification [33,34,35,36,37,38] (Table S1), whether through laboratory analysis of collected samples [39,40,41,42,43,44] or emerging remote monitoring techniques such as ground/airborne sensors, nano sensors, and enzyme-based biosensors [45,46]. The traditional models fail to fully account for the substantial variability in human exposure parameters, which constitutes a major core challenge in health risk assessment. Actual pollutant exposure exhibits significant individual, spatial, and temporal heterogeneity, directly resulting in considerable uncertainty in assessment outcomes based on fixed parameters. Taking the dermal exposure route as an example, key parameters such as skin surface area (SA) and soil–skin adherence factor (AF) vary widely across different populations and studies, which directly impairs the accuracy of dose estimation [47].

These approaches share the other fundamental limitation: they quantify environmental burdens relative to geochemical backgrounds rather than bioavailable exposure. While effective in identifying anthropogenic enrichment, this paradigm fails to distinguish between inert and biologically accessible fractions, potentially overestimating actual risks by assuming 100% bioavailability—a physiologically untenable assumption that neglects the influences of soil matrix properties, metal speciation, and exposure pathways on toxicokinetics [48]. Integrating bioavailability considerations yields dual benefits. For soil remediation, biotechnologies including phytoremediation and microbial remediation substantially reduce costs and environmental disturbances relative to engineering strategies, without generating secondary pollution [49,50]. For health risk assessment, human exposure depends on the fraction solubilized in physiological fluids (e.g., sweat, gastric acid), proportions often considerably lower than total concentrations [51,52,53].

Given escalating environmental pressures, refined health risk assessments that incorporate bioavailability represent an imperative for efficient risk prediction and management.

### 3.3. Advancing Health Risk Assessment Through Bioavailability Integration

Relative to the conventional total-concentration-based risk assessment approaches discussed above, refined health risk assessment, which incorporates individual and spatial heterogeneity as well as bioavailable dose estimation, requires consideration of two critical concepts: bioaccessibility and bioavailability.

Bioaccessibility refers to the fraction of a contaminant that is solubilized and released into simulated physiological fluids (e.g., sweat, gastric juice), representing the maximum pool potentially available for subsequent absorption. This is typically quantified using in vitro assays such as the physiologically based extraction test (PBET) or the unified bioaccessibility method (UBM) [54,55]. Bioavailability, in contrast, denotes the proportion that actually crosses biological barriers, enters systemic circulation, and becomes available to exert systemic toxic effects, a process that often requires in vivo validation (e.g., rat skin perfusion models). In terms of its operational definition, bioavailability requires that contaminants must cross biological membranes [56]. This distinction marks a paradigm shift in health risk assessment [57], moving beyond total concentration measurements to account for site-specific physicochemical interactions governing metal mobilization and uptake.

However, accurately predicting bioavailability is challenging due to its dependence on complex, site-specific interactions between contaminants, environmental matrices, and physiological conditions. To address this, achieving physiologically relevant predictions demands sophisticated modeling frameworks that integrate multi-faceted evidence: (1) Regulatory adaptations (e.g., United States Environmental Protection Agency absorption coefficients for organ-specific uptake) [18]; (2) toxicokinetic studies using in vivo mammalian models to track tissue accumulation [58,59]; and (3) human-relevant platforms like 3D skin organoids that replicate barrier function [60].

As the body’s primary environmental interface, the skin exemplifies the critical role and complexity of bioavailability assessment. It experiences direct exposure, and its barrier function dynamically modulates systemic uptake. The following sections will dissect these dermal bioavailability mechanisms, utilizing the framework above, to establish a foundation for precision risk management.

## 4. Exposure from Models to Scenarios: Practical Pathways for Skin Health Risk Assessment

Heavy metal (HM) exposure poses significant dermatological risks, particularly for high-contact populations. Epidemiological and toxicological studies consistently demonstrate that dermal HM transfer contributes substantially to carcinogenic outcomes, with Cd and Ni identified as high-priority carcinogens through combined ingestion and dermal exposure routes [61]. Population-level correlations link environmental HM concentrations to specific dermatoses: atopic dermatitis incidence correlates with ambient metal levels [62], while psoriasis severity associates with urinary HMs [63], implicating metals in inflammatory pathway dysregulation. These epidemiological patterns are mechanistically investigated through tiered experimental models—including murine in vivo systems and human-relevant 3D skin constructs—which quantify permeation kinetics and resolve molecular pathogenesis.

### 4.1. Health Risk Assessment Models for Skin Exposure

**Figure 1:**
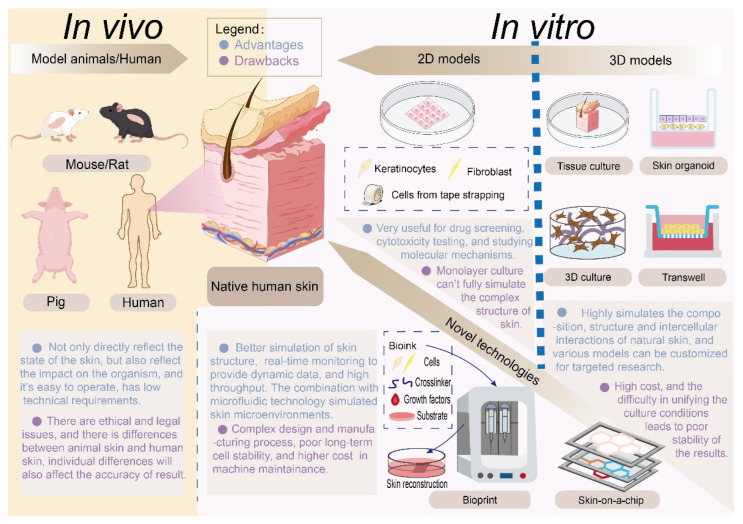
Evolution of in vitro skin models for quantifying dermal bioavailability of heavy metals.

Conventional epidemiological hazard identification requires observable health outcomes, but predictive risk assessment necessitates mechanistic models bridging exposure and toxicity. While clinical patch testing identifies metal sensitization, traditional mammalian models face ethical constraints. Consequently, advanced in vitro alternatives have emerged as critical tools. Reconstructed human epidermis accurately simulates barrier function and absorption dynamics [64]. These systems, summarized in Figure 1, provide indispensable insights into HM bioaccessibility and tissue-specific toxicity, enabling quantitative risk assessment without reliance on clinical manifestation.

Figure 1 illustrates the evolution of skin models and provides an intuitive summary of their development, along with the advantages and limitations of each stage. Skin models can generally be divided into two categories: in vivo models and in vitro models. Their development has progressed from traditional in vivo models to in vitro 2D and 3D models, as well as various novel technologies. Their advantages and disadvantages are also presented in the figure. Generally speaking, in vivo models offer unmatched physiological fidelity but carry ethical concerns and exhibit substantial species and individual variance. To address these issues, in vitro 2D models, 3D models, and novel technologies have been developed to better mimic and approximate real human skin. In vitro 2D cultures enable rapid screening but lack sufficient architectural complexity. By comparison, 3D organotypic or organoid systems deliver high biomimicry and customizability, yet often involve higher costs and greater variability. Emerging bioprinting and skin-on-a-chip technologies are further bridging these gaps, enabling in vivo models to be applied in systemic pathophysiology studies, while in vitro platforms serve as effective tools for scalable drug discovery and personalized dermatology.

### 4.2. Reproduction and Simulation of Exposure Scenarios

Accurate risk assessment demands meticulous replication of real-world exposure conditions. Environmentally, soil heavy metals rarely exist in isolation; they coexist with microplastics (MPs), which elevate soil pH and increase dissolved organic carbon (DOC) to enhance cadmium bioavailability [65,66]. In bodily fluids, HMs co-occur with organic contaminants such as flame retardants, creating synergistic effects—cadmium, for instance, amplifies dermal damage induced by 2-ethylhexyldiphenyl phosphate (EHDPP) [67]. Physiological variabilities further complicate predictions, sweat pH fluctuations (4.0–7.5) dramatically alter metal speciation and solubility, notably converting Cr(III) to highly absorbable Cr(VI) under acidic conditions [14]. Atopic dermatitis (AD) patients exhibit compromised skin barrier function [68]. Age-related barrier thinning is also critical; neonatal stratum corneum and dermis are 20–30% thinner than adult skin, while elderly individuals (>60 years) show a mean thickness of 45.6 µm versus 75.0 µm in younger adults (0–60 years) [69]. Human skin is not a homogeneous tissue, and its barrier function exhibits significant anatomical site-dependent differences. The stratum corneum (SC) is generally thinner in sun-exposed areas (e.g., the face and posterior neck), containing approximately 9–10 cell layers, whereas it is markedly thickened in regions subjected to mechanical stress (e.g., palms and soles), with cell layers exceeding 50 [70]. Sebaceous gland density and secretory activity also show distinct regional variations: sebum production is vigorous in sebum-rich areas (e.g., the face, chest, and back), while sebaceous glands are nearly absent in sites such as the palms, soles, and extensor forearms. Notably, sebum secretion is significantly negatively correlated with skin surface pH—high-sebum areas (e.g., facial skin, pH 4.4–5.6) are acidic, whereas low-sebum regions (e.g., the trunk, pH 5.5–6.5) are relatively alkaline [71,72]. Given that an acidic microenvironment can enhance the solubility of heavy metal ions, and in vitro studies have confirmed that sweat-sebum mixtures further promote the percutaneous penetration of certain metals [14], quantitative assessment of skin exposure risk must explicitly consider anatomical site specificity.

These discrepancies call for the specific dermal exposure models for susceptible populations—accepting population labels as inputs, weighting parameters such as trans epidermal water loss (TEWL), and sweat pH, and generating individualized dose–response functions—is an essential technical avenue for integrating susceptible groups into precision risk management. Future models must incorporate four critical dimensions: (1) pathophysiological relevance (disease-state skin), (2) environmental co-pollutant interactions, (3) population-specific exposure kinetics, and (4) dynamic physicochemical parameters (pH, sebum composition). This multidimensional approach will bridge the gap between controlled laboratory simulations and real-world toxicity manifestations.

Building upon the modeling of exposure scenarios and the reconstruction of dermal contact, a critical bridge has been established linking environmental heavy metal concentrations to potential skin surface doses. However, to achieve the goal of precision health risk assessment, it is imperative to delve deeper into the mechanisms of how these metals breach the skin barrier and the molecular pathways through which they induce damage upon entering tissues. Therefore, the following sections will systematically elucidate the transdermal penetration routes of heavy metals and their key cutaneous toxicological mechanisms, thereby clarifying the biological processes that translate external exposure into internal health effects.

## 5. Transdermal Penetration Mechanisms and Cutaneous Toxicity of Heavy Metals

To systematically elucidate the mechanisms underlying heavy metal-induced dermal toxicity, we propose a hierarchical cascade framework that integrates exposure dynamics, cellular perturbations, and systemic pathological outcomes. This framework comprises three interconnected tiers: (1) the foundational tier of exposure dose, governed by skin permeation; (2) the core tier of cellular and molecular events, encompassing oxidative stress, mitochondrial dysfunction, DNA damage, and programmed cell death pathways; (3) the pathological consequences, manifesting as immune dysregulation, accelerated cutaneous aging, pigmentary disorders, and potential carcinogenesis. The following sections dissect each tier of this cascade, with particular emphasis on the mechanistic transitions from molecular insult to clinical manifestation, to establish a mechanistic foundation for precision dermal risk management.

### 5.1. Transdermal Penetration Mechanisms of Heavy Metals

Human skin consists of stratified structures—stratum corneum (SC), viable epidermis, dermis, and hypodermis—with its appendages (hair follicles and sweat glands) serving as permeation shunts [73]. Corneocytes embedded in lipid matrices constitute the primary barrier [74]. Heavy metals dissolved in sweat adhere to the surface of the stratum corneum, enabling prolonged contact and potential permeation [15]. The permeation of heavy metals mainly occurs through three pathways: lipophilic heavy metals undergo transcellular diffusion across corneocyte membranes [75]; metal ions perform intercellular diffusion through lipid bilayers in accordance with Fick’s law of concentration gradients [11]; and metal particles primarily bypass the stratum corneum through shunts such as hair follicles, undergoing shunt diffusion [76].

Cellular transport systems regulate the intracellular flux of heavy metals: Divalent Metal Transporter 1 (DMT1) is responsible for the influx of divalent metals, ferritin sequesters excess iron [77], and Copper Transporter 1/Copper-transporting P-type ATPase (CTR1/ATP7B) modulates copper homeostasis [78]. Dysregulation of these transporters disrupts intracellular metal equilibrium, thereby triggering cytotoxic cascades, such as oxidative stress and mitochondrial damage (Section 5.2).

Quantitatively, the permeability coefficient (K_p_) measured by Franz diffusion cells can characterize the transdermal flux of heavy metals [79,80]. Since the Franz model is a classic model for studying percutaneous absorption, we collected K_p_ values and uptake flux for several HMs based on this model (Table S2). Evidently, even under the same exposure conditions—identical experimental apparatus, identical sweat pH, and identical metal speciation—the Kp values and bioaccessibility still change significantly with soil properties such as soil pH and matrix composition. This study strongly recommends validating K_p_ values using 3D reconstructed human epidermis (RhE) models and considering the correction of sweat flow rate in dynamic exposure simulations. The critical modifying factors affecting the percutaneous permeation of heavy metals include: barrier integrity (compromised skin increases permeability by 3-5 folds [81]), and metal speciation (Cr(III) oxidizes to more absorbable Cr(VI) in sweat [10]).

Although the percutaneous absorption flux of ions such as nickel, cobalt (Co), and palladium is typically low (in the order of ng/cm^2^/h) [11], a previous study simulating the lead exposure environment of brass foundry workers estimated that, even under low-exposure scenarios, percutaneous absorption could increase blood lead levels by 3.3–6.3 µg/dL [82]. This indicates that chronic exposure leads to significant cutaneous accumulation of heavy metals and induces local toxicity, which is usually undetectable in systemic circulation until pathological manifestations appear. Thus, understanding the mechanisms of skin damage led by HMs is a great beginning for developing effective strategies to mitigate the adverse effects of heavy metal exposure on skin health.

### 5.2. Mechanisms of Heavy Metal-Induced Cutaneous Toxicity

As mentioned above, heavy metals can penetrate the skin barrier through multiple exposure pathways. This section systematically summarizes the series of toxic mechanisms induced in the skin following percutaneous penetration of common heavy metal pollutants in soil. These mechanisms mainly include: the induction of oxidative stress and skin barrier dysfunction, the activation of programmed cell death pathways, the triggering of immunotoxicity and inflammatory responses, the generation of genotoxicity and cellular senescence, as well as the disruption of pigmentation.

**Figure 2:**
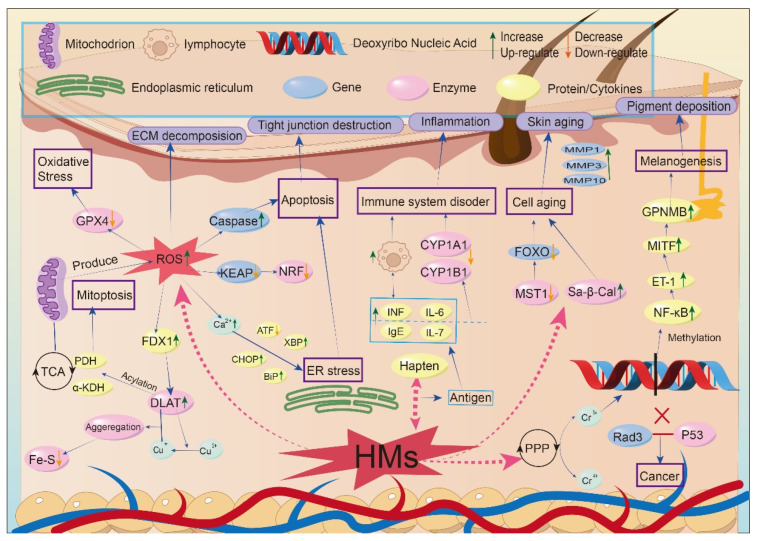
The mechanism by which heavy metals damage the skin.

Given the complexity and interconnection of these mechanisms, Figure 2 was developed to provide a systematic overview. It intuitively illustrates how heavy metals cause molecular and cellular damage within the skin, which ultimately progresses into observable phenotypic manifestations of skin diseases (e.g., extracellular matrix decomposition, tight junction destruction, inflammation).

#### 5.2.1. Oxidative Stress and Barrier Dysfunction

Heavy metals (HMs) disrupt redox homeostasis by generating excessive reactive oxygen species (ROS), which overwhelm the capacity of endogenous antioxidant defense systems. This oxidative imbalance subsequently induces mitochondrial dysfunction, alters cell membrane permeability, and impairs cellular repair mechanisms, thereby establishing a pathological foundation for carcinogenesis [83]. Numerous heavy metals associated with soil pollution, such as Cr, Cd, Zn, Pb, and Cobalt (Co), have been confirmed to induce a state of oxidative stress in cells [84,85,86].

From the perspective of barrier function, ROS degrades hyaluronic acid—a key component of the ECM—compromising skin structural integrity and triggering inflammatory cascades [87]. Hexavalent chromium (Cr(VI)) exemplifies this mechanism: it elevates intracellular Ca^2+^ levels, upregulates endoplasmic reticulum (ER) stress markers Glucose-Regulated Protein 78 (GRP78, also known as Bip) and X-box Binding Protein 1 (XBP-1), while simultaneously inhibiting the stress response mediated by Activating Transcription Factor 6 (ATF6) [88,89]. Subsequent experiments utilizing ATF6 inhibitors confirmed that ROS generated by Cr(VI) degrade tight junctions in human keratinocytes (HaCaT), directly linking this process to skin barrier dysfunction [90]. Impairment of the skin barrier not only induces skin diseases but also further enhances the penetration of pollutants.

#### 5.2.2. Programmed Cell Death Pathways

Programmed cell death is a process regulated by specific genetic mechanisms within cells, which facilitates the elimination of damaged, aged, or superfluous cells. Heavy metals can activate distinct cell death modalities, thereby disrupting tissue homeostasis.

A major mechanism is oxidative stress. Cadmium initiates classical apoptosis via ER-stress gene upregulation (XBP-1, BiP homologous protein) in keratinocytes [91], while Cr(VI) triggers mitochondrial apoptosis through ROS-mediated caspase activation [90]. Excessive manganese (Mn) induces the levels of reactive oxygen species (ROS) and malondialdehyde (MDA), inhibits the activity of manganese superoxide dismutase (Mn-SOD) and glutathione peroxidase (GSH-Px), ultimately leading to cell apoptosis [92].

Although Cu and iron (Fe) are essential metal elements for the human body, excessive accumulation of Cu can induce cuproptosis—a novel cell death mechanism driven by the reduction in Cu^2+^ to Cu^+^ mediated by ferredoxin 1 (FDX1) Cu^+^ binds to the lipoylated dihydrolipoamide S-acetyltransferase (DLAT) protein, inhibits the assembly of iron-sulfur clusters (Fe-S clusters), and leads to the toxic aggregation of tricarboxylic acid (TCA) cycle enzymes [93,94]. In contrast to cuproptosis, iron-dependent ferroptosis is induced by the inhibition of glutathione peroxidase 4 (GPX4). Both of these cell death modalities are implicated in the pathogenesis of psoriasis, eczema, and melanoma [95,96,97,98]. Thus, understanding the pollution mechanisms of heavy metals may not only provide new ideas for diseases with unknown etiologies but also arouse public attention to the hazards of heavy metal pollution.

#### 5.2.3. Immunotoxicity and Inflammation

Heavy metals (HMs) function as haptens that conjugate with cutaneous proteins to form complete antigens, activating Langerhans cells and initiating type IV hypersensitivity reactions via the Gell–Coombs pathway [99]. Such immunotoxicity does not result from a single mechanism but arises jointly through multiple interconnected molecular pathways, exerting profound effects on skin health.

As the most common sensitizing metal, nickel has a representative pathogenic mechanism. Nil binds toll-like receptor 4 (TLR4), triggering IgE-mediated hypersensitivity reactions, and it upregulates the inflammatory cytokines (e.g., interleukin-8 and interleukin-18), thereby driving allergic contact dermatitis (ACD) [100,101]. A study demonstrated that sensitization to Ni^2+^ in mice occurs independently of TLR4, MyD88 (Myeloid differentiation primary response 88)-dependent, and IL-1 (interleukin-1)-associated pathways that mediate the immune response to Ni^2+^ in their in vivo model [102]. This suggests that alternative or redundant immune activation pathways may underlie heavy metal sensitization.

Different heavy metals disrupt specific immune balances, thereby leading to cutaneous diseases of distinct natures. Arsenic exposure elevates serum IgE (Immunoglobulin E) and disrupts Th1 (T helper1 cell)/Th2 (T helper2 cell) lymphocyte balance through STAT6 (signal transducer and activator of transcription 6) hyperactivation, promoting atopic dermatitis pathogenesis [62,103]. In contrast, cadmium amplifies pro-inflammatory cytokines, including TNF-α (Tumor Necrosis Factor-alpha), IFN-γ (Interferon-gamma), and IL-17 (Interleukin-17), expanding CD4^+^/CD8^+^ T-cell (T lymphocyte) populations and exacerbating contact hypersensitivity reactions [104]. Concurrently, arsenic and mercury suppress cytochrome P450 enzymes (CYP1A1, CYP1B1) [105,106]. Fibroblast studies confirm HM-laden dust exposure upregulates IL-6 (interleukin-6) and TNF-α expression 3–5-fold via NF-κB (Nuclear Factor kappa-light-chain-enhancer) hyperactivation, fundamentally disrupting cutaneous immune equilibrium [107].

Thus, heavy metals damage the cutaneous immune system through the above mechanisms and exert dual toxicity on the skin: they not only impair the body’s detoxification capacity against exogenous toxic substances, but also disrupt immune homeostasis and trigger persistent inflammatory responses, further compromising skin barrier function and immune defense.

#### 5.2.4. Genotoxic and Senescent Effects

Heavy metal-induced genomic instability and accelerated skin aging constitute the core mechanisms underlying their long-term cutaneous toxicity. This process is not a simple superposition of isolated events, but a cascade ranging from genetic damage to comprehensive decline in cellular function, which profoundly reveals the intrinsic logic by which heavy metal exposure increases the risk of premature skin aging and carcinogenesis.

Heavy metals induce genomic instability through direct Deoxyribonucleic acid (DNA) interactions and epigenetic alterations, fundamentally disrupting the genetic stability of cells. Chromium (Cr(VI)) undergoes mitochondrial reduction to Cr(V), generating hydroxyl radicals that cause double-strand breaks while inactivating the tumor suppressor p53 and inhibiting DNA repair via Rad3 kinase (ataxia telangiectasia and Rad3-related) suppression [108]. Arsenic promotes p53 hypermethylation, impairing its transcriptional activity and enabling uncontrolled proliferation in epithelial tissues [109]. Cadmium exposure induces phosphorylation of histone H2AX (H2A histone family member X), a biomarker of DNA breakage, while disrupting nucleotide excision repair mechanisms [91,110].

Such genetic damage ultimately manifests as accelerated aging of skin tissue. Chronic exposure accelerates skin aging through interconnected pathways: Telomeres shorten in keratinocytes, inducing replicative senescence [111]. Arsenic suppresses the MST1-FOXO (Macrophage Stimulating 1- Forkhead Box O) signaling axis in fibroblasts, triggering irreversible growth arrest marked by elevated senescence-associated β-galactosidase (SA-β-Gal) activity [112]. Senescent cells adopt a secretory phenotype (SASP), releasing matrix metalloproteinases (MMP-1, MMP-3, MMP-10) that degrade collagen and elastin networks, reducing skin elasticity by >50% [113]. Finally, mitochondrial dysfunction further exacerbates aging; HM-generated ROS deplete NAD^+^ (Nicotinamide adenine dinucleotide) reserves, impairing sirtuin-mediated maintenance of proteostasis and metabolic homeostasis [114].

In summary, heavy metals initiate a well-defined skin aging pathway spanning from the molecular to tissue level, starting with the induction of DNA damage and epigenetic disorders, triggering cellular senescence programs, and ultimately disrupting the skin extracellular matrix via the SASP. Understanding this complete pathway provides key theoretical targets for developing targeted intervention strategies, such as enhancing DNA repair, eliminating senescent cells, or preserving mitochondrial function.

#### 5.2.5. Pigmentation Disorders

Heavy metal exposure induces dysregulated melanogenesis through multifaceted molecular pathways. That process not only reveals an important clinical manifestation of heavy metal-induced cutaneous toxicity, but also elucidates at the molecular level how environmental pollutants disrupt cellular homeostasis.

Firstly, different heavy metals trigger pigmentation through distinct initiation mechanisms. Iron deposition in dermal tissues directly promotes hyperpigmentation by catalyzing melanin synthesis [115], while arsenic-driven pigmentation—a clinical hallmark of chronic poisoning—involves epigenetic reprogramming through altered DNA methylation and sustained activation of the NF-κB pathway [116]. In the inflammatory context initiated by heavy metals such as arsenic, NF-κB activation transcriptionally upregulates endothelin-1 (ET-1) [117], which initiates a melanogenic cascade through paracrine signaling. ET-1 binds to endothelin receptors on melanocytes, triggering the microphthalmia-associated transcription factor (MITF) to transactivate glycoprotein non-metastatic melanoma protein B (GPNMB)—a key regulator of melanosome maturation and pigment transfer [118].

Concurrently, mitochondrial calcium flux modulates pigment production. The mitochondrial calcium uniporter (MCU) enhances melanogenesis by elevating intramitochondrial Calcium ion (Ca^2+^), which stimulates rate-limiting enzymes in melanin biosynthesis [119]. Notably, heavy metal-induced inflammation increases cytosolic Ca^2+^ concentrations, potentially amplifying MCU-mediated melanogenesis. However, direct mechanistic evidence linking inflammatory Ca^2+^ surges to MCU activation in pigmentary disorders remains limited, representing a critical knowledge gap.

These interconnected pathways—spanning epigenetic dysregulation, cytokine signaling, and organellar ion dynamics—collectively drive pathological hyperpigmentation. Understanding this networked mechanism not only clarifies the importance of heavy metals in causing cutaneous pigmentary disorders but also provides potential theoretical targets for preventing or treating such pollution-related skin diseases by blocking specific steps—such as inhibiting inflammation with antioxidants, regulating calcium signaling, or modulating MITF activity.

## 6. Precision Control Strategies for Heavy Metal Pollution in Urban Soils

To translate exposure reduction strategies into enforceable standards, dermal occupational exposure limits (DOELs) and Derived No-Effect Levels (DNELs) were proposed for occupational or consumer scenarios [120,121]. To reduce risks of HMs, preventing pollution with physicochemical and biological soil-remediation strategies remains the most effective strategy, yet there is still much that individuals can do on their own to further minimize exposure. Since heavy metals penetrate skin slowly, frequent hand–washing [122] and wearing gloves in contaminated areas [123] are effective preventive measures.

Dietary choices also play a role. Antioxidant-rich foods like grapes can reduce DNA damage markers and oxidative stress caused by heavy metals [124]. Sulforaphane (SFN) can suppress melanin-related signals and inflammatory cytokines [125], while Vitamin E protects against copper-induced dermatitis [126], and Vitamin C alleviates heavy metal-induced oxidative stress, particularly for lead poisoning [127]. Nutrient intake can influence heavy metal absorption. Iron deficiency increases lead and cadmium availability [128]. Calcium supplementation reduces cadmium availability [129] and lowers lead, cadmium, and arsenic availability in mice [130].

In summary, a feasible pathway to reduce heavy metal exposure risks constitutes a comprehensive chain system that spans from macro-level policy controls to micro-level individual behaviors. It begins with the establishment of national standards, relies on the selection of scientific monitoring and remediation technologies, and ultimately achieves precise prevention and control through enhancing individual risk awareness and protective capabilities, thereby effectively safeguarding both the ecological environment and public health security.

## 7. Conclusions

In summary, urban green spaces (UGSs) are of great value in improving environmental quality and safeguarding public health. However, their soils are increasingly threatened by heavy metal contamination, presenting a dilemma between maximizing the ecological benefits of UGS and minimizing associated contamination risks. This paper systematically reviews the sources, spatial distribution, and ecological and health risks of heavy metals in urban green space soils. Existing studies show that traditional health risk assessment methods based solely on total heavy metal concentrations have obvious limitations: they fail to consider the variability in heavy metal bioavailability and the dynamics of the skin microenvironment, and tend to overestimate actual health risks.

To address these shortcomings, this review proposes a more refined health risk assessment framework that incorporates heavy metal bioaccessibility and dermal absorption kinetics. Meanwhile, this paper objectively identifies the main limitations of current research. First, uncertainties remain in extrapolating from toxicological mechanisms to population-level risks. Most available molecular toxicological evidence is derived from in vitro experiments or animal models, and their validity for simulating long-term, low-dose, and combined exposure scenarios in real human populations requires further verification. Second, the practical application of refined risk assessment faces both technical and data challenges. As a key parameter, bioavailability exhibits strong spatiotemporal heterogeneity, and cost-effective real-time in situ monitoring networks are still lacking to support dynamic assessment. Furthermore, quantifying and integrating susceptible factors such as individual genetics and health status (e.g., skin barrier function) into predictive models remains a critical bottleneck.

Despite these limitations, this study provides a more refined perspective for the risk management of urban soil pollution and facilitates the development of targeted mitigation strategies to effectively protect public health. Integrating bioavailability into the risk assessment framework can avoid the waste of remediation resources caused by “over-assessment” and accurately identify contamination scenarios that pose actual health risks, thus offering a more cost-effective technical approach for health risk management of heavy metals in urban soils. Future research should further strengthen the integration of human biological data and risk prediction models, and establish an integrated “monitoring–prediction–intervention” system to consolidate the health barrier for the sustainable development of urban human settlements.

## Data Availability

No new data were created or analyzed in this study. Data sharing is not applicable to this article.

## Supplementary Materials

The following supporting information can be downloaded at: https://www.mdpi.com/article/10.3390/toxics14030236/s1. Table S1: Assessment results of heavy metals in urban soils in various regions. Table S2. Summary of heavy metal dermal penetration parameters based on the Franz model.
