# Supplementary material for: Dermal Exposure to Heavy Metals in Urban Green Space Soils: A Review of Bioavailability, Toxic Mechanisms, and Precision Risk Assessment
Source: Toxics. 2026 Mar 10;14(3):236. doi: 10.3390/toxics14030236 (PMC13030796; doi:10.3390/toxics14030236)
Supplement: Supplementary file 1 [file toxics-14-00236-s001.zip › toxics-4150692-supplementary.pdf]

Table S1. Assessment results of heavy metals in urban soils in various regions

	Region	Sampling Location	Instrument	Average Heavy Metal Contents(mg/kg)						Assessment Method	Assessment Result	References
				Cu	Cr	Cd	Zn	Pb	Ni			
1	Beijing, China	Residential areas	ICP-MS	22	90.7	0.3	80.6	54	30.9	The risk assessment model recommended by USEPA	The health risks of the soil were negligible.	[1]
2	Shandong, China	City proper	ICP-MS	18.7	48.4	0.2	48.9	31.0	20.6	IMN, RI	Hg had the most severe health risk.	[2]
3	Hubei, China	Urban parks	ICP-MS	31.4	58.7	2.5	186.3	34.9	27.0	Monte Carlo health risk evaluation model	Cd and Cr in children were a concern for cancer risks.	[3]
4	Shanghai, China	Roadside trees	ICP-AES	28.6	80.8	0.1	131.8	25.5	32.0	Hakanson's risk evaluation method	Cd exhibited the highest potential ecological risk.	[4]
5	Neimeng -gu, China	City proper	ICP-MS	24.0	70.3	0.2	109.7	48.4	26.7	RI, *Igeo*	Hg, Pb, Zn, and Cu have higher pollution levels	[5]
6	Jiangsu, China	City proper	ICP-AES	25.5	72.9	0.5	90.2	37.6	38.2	HRI	Cd represented a high-risk state.	[6]
7	Guangdong, China	City parks	ICP-MS	44.0	51.7	0.6	203.0	105.0	19.9	*Igeo*, IMN, mCd, CSI	Most samples had high ecological risk.	[7]
8	Jilin,China	City proper	pXRF	43.8	64.2	/	96.2	57.4	/	PI, EF, *Igeo*, Er Nemerow Index, PLI	As had a relatively higher ecological risk.	[8]

9	Zhejiang, China	City and suburban	ICP-MS	32.5	63.6	0.3	125.0	53.7	26.5	PLI, *Igeo*	No significant heavy metal pollution.	[9]
10	Vienna, Austria	City park	ICP-OES	49.0	15.0	1.4	130.0	79.0	20.0	PI	Cd had a moderate level of pollution.	[10]
11	Karachi, Pakistan	Roadsides, industrial, and residential areas	AAS	33.3	9.6	0.3	99.5	42.1	9.4	EF, *Igeo*, HHRA	Carcinogenic and noncarcinogenic-Genetic risk was insignificant.	[11]
12	Ufa city, Russia	City areas	INAA,XRF	51.1	346.0	/	150.0	45.4	101.0	EF, *Igeo*, CC	Cr and Ni were identified as pollution elements.	[12]
13	Cluj-Napoca, Romania	Public playgrounds	XRF	26.75	35.0	/	106.9	32.1	33.5	PLI	Zn, Pb, As, and Mn contributed most to the PLI.	[13]
14	Grand Forks, America	Residential areas	ICP-MS	17.4	23.6	0.7	81.6	14.7	24.8	CF, *Igeo*	As and Cr posed Significant risks to populations.	[14]
15	Katima Mulilo, Namibia	Urban motor park	ICP-OES	5.0	2.4	0.5	1.0	8.9	1.7	RI	Cd recorded the highest ecological risk indices.	[15]
16	Ancona, Italy	Recreational areas, and gardens	ICP-MS, XRF	/	45.5	/	158.5	64.0	50.0	Extractability, ToA	Pb and Zn warrant greater attention.	[16]

Annotation: AAS (Atomic Absorption Spectroscopy), CC (Concentration coefficients), CF (Contamination factor), CSI (Contamination security index), EF (enrichment factor), Er (Ecological Risk Factor), HHRA (Human Health Risk Assessment), IMN (Improved Nemerow index), ICP-AES (Inductively Coupled Plasma - Atomic Emission Spectrometry), ICP-MS (Inductively Coupled Plasma - Mass Spectrometry), ICP-OES (Inductively Coupled Plasma - Optical Emission Spectrometry), INAA (Instrumental Neutron Activation Analysis), mCd (degree of contamination), PI (Pollution Index), PLI (Geoaccumulation index), RI (Ecological risk indexes), pXRF (Portable X-ray Fluorescence Spectrometer), XRF (X-ray Fluorescence Spectrometer), ToA (Threshold of concern for the definition of extractability).

Table S2. Summary of heavy metal dermal penetration parameters based on the Franz model

	Compound	Average uptake flux ($gcm^{-2}h^{-1}$)	K_p (cm/h)	Exposure model	Exposure solution	Exposure period (h)	References
1	Chromium		4.7×10^{-2} to 1.1×10^{-1}	Franz model with synthetic membrane	Soil extract with artificial sweat (pH=6.5)		[17]
2	Chromium		1.7×10^{-3} to 2.3×10^{-3}	Franz model with a Strat-M® membrane	Soil extract with artificial sweat (pH=6.5)		[18]
3	Chromium		4×10^{-2} to 1.3×10^{-1}	Franz model with a Strat-M® membrane	Soil extract with artificial sweat (pH=6.5)		[19]
4	Copper		2.4×10^{-2} to 5.2×10^{-1}				
5	Cobalt	$1.2\times 10^{-5} \pm 0.0054$		Franz model with human abdominal full-thickness skin	Metal powder extract with artificial sweat (pH=6.5)	24h	[20]
6	Nickel	$1.7\times 10^{-5} \pm 0.00036$					
7	Lead		4.0×10^{-2}	Franz model with human abdominal flank full-thickness skin	Road dust powder extract with artificial sweat (pH=6.5)		[21]
8	Chromium		1.3×10^{-2}				
9	Cobalt		1.0×10^{-3}				
10	Nickel		8.0×10^{-3}				

Annotation: Kp (Permeation Coefficient), Strat-M® membrane (a synthetic, non-animal model for in vitro transdermal diffusion studies, exhibits negligible inter-batch variability, and enabling prediction of permeation across human skin).

Reference

1. Peng, C.; Zhang, K.; Wang, M.; Wan, X.; Chen, W. Estimation of the accumulation rates and health risks of heavy metals in residential soils of three metropolitan cities in China. J. Environ. Sci. 2022, 115, 149–161.
2. Cao, B.; Sun, Z.; Bai, D.; Kong, L.; Zhang, X.; Chen, J.; Chen, D. The Identification of Soil Heavy Metal Sources and Environmental Risks in Industrial City Peri-Urban Areas: A Case Study from a Typical Peri-Urban Area in Western Laizhou, Shandong, China. *Sustainability* **2024**, *16*, 4655.
3. Sun, J.; Chen, M.; Xiao, J.; Xu, G.; Zhang, H.; Zhang, G.; Yang, F.; Zhao, C.; Guo, L. Exploring the Spatial Distribution Characteristics of Urban Soil Heavy Metals in Different Levels of Urbanization. *Agronomy* **2025**, *15*, 418..
4. He K.; Wang J.; Geng, H.; Qin, Z.; Li, N.; Zhang, Y.; Yang, R.; Feng, S.; Wang, B.. Will different land uses affect heavy metal pollution in soils of roadside trees? An empirical study from Shanghai. *Environmental Monitoring and Assessment*, **2023**, 195(11): 1388.
5. Chen, X.; Ren, Y.; Li, C.; Shang, Y.; Ji, R.; Yao, D.; He, Y. Pollution Characteristics and Ecological Risk Assessment of Typical Heavy Metals in the Soil of the Heavy Industrial City Baotou. *Processes* **2025**, *13*, 170.
6. Li, Y.; Dong, Z.; Feng, D.; Zhang, X.; Jia, Z.; Fan, Q.; Liu, K. Study on the risk of soil heavy metal pollution in typical developed cities in eastern China. *Sci. Rep.* **2022**, *12*, 3855.
7. Zhou, L.; Wang, S.; Hao, Q.; Kang, L.; Kang, C.; Yang, J.; Yang, W.; Jiang, J.; Huang, L.-Q.; Guo, L. Bioaccessibility and risk assessment of heavy metals, and analysis of arsenic speciation in Cordyceps sinensis. *Chin. Med.* **2018**, *13*, 40. https://doi.org/10.1186/s13020-018-0196-7.
8. Zou, X.; Lu, J.; Zhao, X.; Wei, Q.; Gou, Z.; Hou, Y.; Lai, Y. An Investigation into the Viability of Portable Proximal Sensor X-Ray Fluorescence Data for Assessing Heavy Metal Contamination in Urban Soils: A Case Study in Changchun, China. *Toxics* **2024**, *12*, 798.
9. Zhang, P.; Hu, L.; Gao, B.; Gao, F.; Zhu, X.; Li, Y.; Yao, H. Spatial–temporal variation and source analysis of heavy metals in different land use types in Beilun District (2015 and 2022). *Sci. Rep.* **2024**, *14*, 15127.
10. Bibi, D.; Tőzsér, D.; Sipos, B.; Tóthmérész, B.; Simon, E. Heavy Metal Pollution of Soil in Vienna, Austria. *Water Air Soil Pollut.* **2023**, *234*, 232.
11. Karim, Z.; Qureshi, B.A. Health Risk Assessment of Heavy Metals in Urban Soil of Karachi, Pakistan. *Hum. Ecol. Risk Assess. Int. J.* **2014**, *20*, 658–667.
12. Goncharov, G.; Soktoev, B.; Farkhutdinov, I.; Matveenko, I. Heavy metals in urban soil: Contamination levels, spatial distribution and human health risk assessment (the case of Ufa city, Russia). *Environ. Res.* **2024**, *257*, 119216.
13. Răcușan Ghircoiaș, O.; Tănăselia, C.; Chintoanu, M.; Crișan, I.; Hoble, A.; Ștefan, R.; Dîrja, M. Relevance of Soil Heavy Metal XRF Screening for Quality and Landscaping of Public Playgrounds. *Toxics* **2023**, *11*, 530.
14. Saleem, M.; Sens, D.A.; Somji, S.; Pierce, D.; Wang, Y.; Leopold, A.; Haque, M.E.; Garrett, S.H. Contamination Assessment and Potential Human Health Risks of Heavy Metals in Urban Soils from Grand Forks, North Dakota, USA. Toxics 2023, 11, 132. https://doi.org/10.3390/toxics11020132.
15. Abah J, Simasiku E K, et al. Assessment of heavy metals pollution status of surface soil dusts at the Katima Mulilo urban motor park, Namibia. *Geomatics, Natural Hazards and Risk*, 2023, 14(1).
16. Serrani, D.; Ajmone-Marsan, F.; Corti, G.; Cocco, S.; Cardelli, V.; Adamo, P. Heavy metal load and effects on biochemical properties in urban soils of a medium-sized city, Ancona, Italy. Environ. *Geochem. Health* **2022**, *44*, 3425–3449. https://doi.org/10.1007/s10653-021-01105-8.
17. Marin Villegas, C.A.; Zagury, G.J. Comparison of Synthetic Sweat and Influence of Sebum in the Permeation of Bioaccessible Metal(loid)s from Contaminated Soils through a Synthetic Skin Membrane. *Environ. Sci. Technol.* **2021**, *55*, 8215–8222. https://doi.org/10.1021/acs.est.1c02038.
18. Ghislain, F.A.; Zagury, G.J. Influence of sebum proportion in synthetic sweat on dermal bioaccessibility and on permeation of metal(loid)s from contaminated soils. *Environ. Sci. Pollut. Res.* **2023**, *30*, 86762–86772. https://doi.org/10.1007/s11356-023-28388-x.
19. Marin Villegas, C.A.; Zagury, G.J. Metal(loid) speciation in dermal bioaccessibility extracts from contaminated soils and permeation through synthetic skin. J. *Hazard. Mater*. **2023**, *455*, 131523. https://doi.org/10.1016/j.jhazmat.2023.131523.
20. Larese, F.; Gianpietro, A.; Venier, M.; Maina, G.; Renzi, N. In vitro percutaneous absorption of metal compounds. *Toxicol. Lett.* **2007**, *170*, 49–56.
21. Magnano, G.C.; Marussi, G.; Pavoni, E.; Adami, G.; Larese Filon, F.; Crosera, M. Percutaneous metals absorption following exposure to road dust powder. *Environ. Pollut.* **2022**, *292,* 118353. https://doi.org/10.1016/j.envpol.2021.118353.
